# Supplementary material for: From sequence to enzyme mechanism using multi-label machine learning
Source: BMC Bioinformatics. 2014 May 19;15:150. doi: 10.1186/1471-2105-15-150 (PMC4229970; doi:10.1186/1471-2105-15-150)
Supplement: Additional file 2 — Java code of ml2db. Additional file ml2db_code.tar.gz contains the Java source code to run the multi-label machine learning experiments and save the results to database. The code’s Javadoc is included. [file 1471-2105-15-150-S2.zip › additional file 2/ml2db/ml2db/doc/mulan/classifier/neural/MMPRandomizedUpdateRule.html]

MMPRandomizedUpdateRule


JavaScript is disabled on your browser.


- Overview
- Package
- Class
- Use
- Tree
- Deprecated
- Index
- Help

- Prev Class
- Next Class

- Frames
- No Frames

- All Classes

- Summary:
- Nested |
- Field |
- Constr |
- Method

- Detail:
- Field |
- Constr |
- Method


mulan.classifier.neural

## Class MMPRandomizedUpdateRule

- java.lang.Object
- - mulan.classifier.neural.MMPUpdateRuleBase
  - - mulan.classifier.neural.MMPRandomizedUpdateRule

- All Implemented Interfaces:
  :   ModelUpdateRule

  ---

    

  ```
  public class MMPRandomizedUpdateRule
  extends MMPUpdateRuleBase
  ```

  Implementation of randomized update rule for `MMPLearner`. It is a randomized variation
  of `MMPUniformUpdateRule`. A opposed to uniformed update, the randomized version will
  penalize each wrongly order pair of labels by random value from interval <0,1>. Afterwards, the
  penalty weights are normalized, so their sum is equal to 1.  
  The model is represented as a list of perceptrons (one for each label), each represented
  by `Neuron`. Perceptrons are expected to be in the same order as labels in training data set.

  Version:
  :   2012.02.27

  Author:
  :   Jozef Vilcek

  See Also:
  :   `MMPUpdateRuleBase`

- - ### Constructor Summary

    Constructors

    Constructor and Description

    `MMPRandomizedUpdateRule(java.util.List<Neuron> perceptrons,
    RankingLossFunction lossMeasure)`

    Creates a new instance of `MMPRandomizedUpdateRule`.
  - ### Method Summary

    - ### Methods inherited from class mulan.classifier.neural.MMPUpdateRuleBase

      `process`
    - ### Methods inherited from class java.lang.Object

      `equals, getClass, hashCode, notify, notifyAll, toString, wait, wait, wait`

- - ### Constructor Detail


    - #### MMPRandomizedUpdateRule

      ```
      public MMPRandomizedUpdateRule(java.util.List<Neuron> perceptrons,
                             RankingLossFunction lossMeasure)
      ```

      Creates a new instance of `MMPRandomizedUpdateRule`.

      Parameters:
      :   `perceptrons` - the list of perceptrons, representing the model, which will receive updates.
      :   `lossMeasure` - the loss measure used to decide when the model should be updated by the rule


- Overview
- Package
- Class
- Use
- Tree
- Deprecated
- Index
- Help

- Prev Class
- Next Class

- Frames
- No Frames

- All Classes

- Summary:
- Nested |
- Field |
- Constr |
- Method

- Detail:
- Field |
- Constr |
- Method
